# Rape-related symptoms in adolescents: short- and long-term outcome after cognitive behavior group therapy

**DOI:** 10.3402/ejpt.v5.22969

**Published:** 2014-06-03

**Authors:** Iva Bicanic, Carlijn de Roos, Floryt van Wesel, Gerben Sinnema, Elise van de Putte

**Affiliations:** 1 National Psychotrauma Center for Children and Youth, University Medical Center Utrecht, Utrecht, The Netherlands; 2 Psychotrauma Center for Children and Youth, GGZ Rivierduinen Leiden, Leiden, The Netherlands; 3 Department of Methodology and Statistics, University of Utrecht, Utrecht, The Netherlands; 4 Department of Pediatrics, University Medical Center Utrecht, Utrecht, The Netherlands

**Keywords:** Adolescents, cognitive behavior therapy, group therapy, PTS symptoms, rape, sexual assault

## Abstract

**Background:**

Efficacy studies on treatment in adolescent victims of single rape are lacking, even though sexual victimization is most likely to occur during adolescence and despite the fact that adolescents are at risk to develop subsequent posttraumatic stress disorder.

**Aim:**

The aim of this prospective observational study was to evaluate the short- and long-term outcomes of a nine-session cognitive behavior group therapy (STEPS), including a parallel six-session parents’ group on rape-related symptomatology in female adolescents (13–18 years). STEPS includes psychoeducation, exposure *in sensu* as well as *in vivo*, cognitive restructuring, and relapse prevention.

**Methods:**

Fifty-five female adolescents with mental health problems due to single rape, but without prior sexual trauma, received STEPS while their parents participated in a support group. Subjects were assessed on posttraumatic stress (PTS) and comorbid symptoms using self-report questionnaires prior to and directly after treatment, and at 6 and 12 months follow-up.

**Results:**

Repeated measures analysis showed a significant and large decrease in symptoms of PTS, anxiety, depression, anger, dissociation, sexual concerns, and behavior problems directly after treatment, which maintained at 12 months follow-up. Time since trauma did not influence the results. Dropout during STEPS was 1.8%.

**Conclusions:**

The results potentially suggest that the positive treatment outcomes at short- and long-term may be caused by STEPS. The encouraging findings need confirmation in future controlled studies on the effectiveness of STEPS because it may be possible that the treatment works especially well for more chronic symptoms, while the less chronic part of the sample showed considerable improvement on its own.

The experience of rape is associated with the development of serious mental health disorders, most commonly *DSM-IV*-defined acute stress disorder and posttraumatic stress disorder (PTSD; Hansen, Armour, & Elklit, 2012; Kessler, Sonnega, Bromet, Hughes, & Nelson, 1995; Rothbaum, Foa, Riggs, Murdock, & Walsh, 1992). The severe consequences of rape emphasize the need for effective treatment. Various types of exposure-based cognitive behavior therapy (CBT; Vickerman & Margolin, 2009) and eye movement desensitization reprocessing (EMDR; Rothbaum, Astin, & Marsteller, 2005) have shown to be effective in reducing PTSD following sexual trauma. Typically, these studies involve adult women. In 8- to 14-year-old children, trauma-focused CBT (TF-CBT) proves to be effective in reducing PTSD caused by sexual trauma (Avinger & Jones, 2007; Deblinger, Mannarino, Cohen, & Steer, 2006), especially when parents are involved in the treatment (Cohen, Deblinger, Mannarino, & Steer, 2004). The majority of the subjects in these studies were victimized by multiple (sexual) traumas often beginning in early life, making it impossible to differentiate between psychopathologies due to early traumas or recent sexual trauma.

Efficacy studies on treatment in adolescent victims of *single* rape are lacking, even though sexual victimization is most likely to occur during adolescence (Humphrey & White, 2000). Also, adolescents are most at risk to develop subsequent PTSD (McLaughlin et al., 2013). Reducing PTSD symptoms is highly important because they negatively interfere with school and social functioning. Moreover, victims are at an increased risk for subsequent sexual assaults when they do not recover from PTSD (Risser, Hetzell-Riggin, Thomsen, & McCanne, 2006).

For adolescent victims of a first rape with no prior sexual trauma, the STEPS cognitive behavior group therapy protocol of nine sessions with a parallel parents’ group of six sessions was developed at the University Medical Center Utrecht (Bicanic & Kremers, 2007a, 2007b, 2007c). STEPS includes psychoeducation, exposure *in sensu* as well as *in vivo*, cognitive restructuring, and relapse prevention. The objective of the present study was to assess rape-related symptomatology, including posttraumatic stress (PTS) symptoms, as well as behavioral problems in adolescent female victims of a single rape both directly after STEPS treatment and at long-term follow-up. We hypothesized that rape-related symptoms would decrease significantly after STEPS, in particular directly after treatment, and that this gain would maintain at long-term follow-up.

## Methods

### Subjects

Between 2005 and 2009, 193 female adolescents aged 13–18 years with rape-related mental health problems were referred to two Dutch Psychotrauma Centers: one in Utrecht and one in Leiden. Referral sources included police, victim advocacy centers, schools, mental health centers, and self-referrals. Rape was defined as an event that involves the use or threat of force to penetrate the victim’s vagina or anus by penis, tongue, fingers, or object, or the victim’s mouth by penis; the definition includes both attempted and completed rape (Tjaden & Thoennes, 2006).

**Fig. 1:**
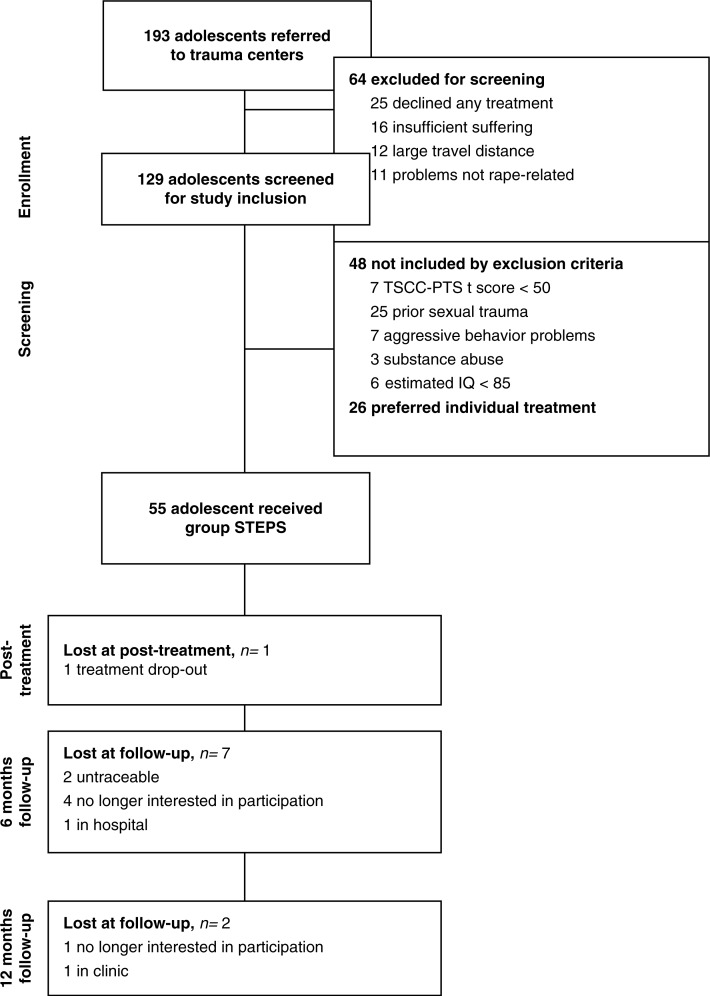
Flowchart of enrolment, screening, and follow-up.

Of the 193 referred adolescents, a total of 129 adolescents were screened for study inclusion. STEPS group therapy was applied in a final set of 55 adolescent girls and their parents. The subject flowchart from enrolment to study and follow-up is presented in Fig. 1.

Inclusion criteria for STEPS group therapy included the experience of rape that occurred on one occasion with one or more perpetrators. Next to the event, a minimum *t*-score of 50 on the subscale, PTS of the Trauma Symptom Checklist for Children (TSCC), defined as TSCC-PTS (Briere, 1996; Dutch translation by Bal, 1998, unpublished data), was required for inclusion into STEPS group therapy, implying minimum subclinical levels of PTS symptoms based on normative data for the TSCC (Briere, 1996). Additionally, at least one parent had to participate in the parallel parents’ group. Subjects were excluded for STEPS group therapy in case of prior sexual trauma, the perpetrator being a first degree family member, a TSCC-PTS *t*-score <50, current extreme disruptive behavior, active substance abuse disorder, active psychosis, estimated IQ level <85 based on school level, or concurrent psychotherapy.

The assessment and treatment protocols were identical at both study sites and were reviewed and approved by the institutional review boards at each center. Informed adolescent and parental consent was required for admission to the study. The STEPS group therapists from Utrecht were the developers of STEPS. They trained the therapists in Leiden.

### Measures

Information about the subjects’ mental health functioning was obtained by self-report questionnaires. Questionnaires were completed before and after treatment, and at 6 and 12 months follow-up. The subjects completed the TSCC (Briere, 1996; Dutch translation by Bal, 1998, unpublished data), a checklist for anxiety, depression, PTS, anger, sexual problems, and dissociation. Data on the TSCC suggest that internal reliability is adequate (Cronbach’s alphas ranging from 0.77 to 0.89) and that convergent and discriminant validity indices are reported to be satisfactory (Bal & Uvin, 2009; Briere, 1996).

Subjects also completed the Youth Self-Report of the Child behavior Checklist (YSR; Achenbach & Rescorla, 2001; Dutch translation by Verhulst, Van der Ende, & Koot, 1997), which evaluates the teenager’s perception of behavioral problems. YSR has shown to be internally reliable (Cronbach’s alphas ranging from 0.71 to 0.95), and convergent and discriminant validity are reported to be satisfactory (Bérubé & Achenbach, 2006). The YSR includes four broadband scales and nine narrowband scales to assess child behavior problems. For the purpose of the study, only the broadband scales of internalizing, externalizing, and total behavior problems were included in the analyses.

Only at baseline, included subjects completed the Symptom Checklist-90-R (SCL-90; Arrindell & Ettema, 1986), a 90-item questionnaire assessing present psychopathology and were evaluated with an assessment interview, including a detailed trauma history, information about the lifetime number and types of trauma, and an evaluation of trauma and perpetrator characteristics. To determine whether the subject experienced prior sexual trauma, the childhood unwanted sexual events list (Lange, 2004) was used.

Also, 37 of the 55 subjects (67%) participated in a neurobiological study (Bicanic et al., 2013). These subjects were administered with the Dutch version of the Anxiety Disorders Interview Schedule—Children’s version (ADIS-C; Siebelink and Treffers, 2001; Silverman and Albano, 1996), a DSM-IV-based, semi-structured clinical interview to determine the presence of PTSD and potential other psychopathology. All the 37 patients were diagnosed with a PTSD diagnosis according to the DSM-IV. We found no significant difference on the TSCC-PTS subscale between those with an interviewer-assessed PTSD (*n*=37) and those without (*n*=18).

### Treatment protocol

STEPS is a cognitive behavior treatment protocol for adolescent girls with rape-related problems and their parents (Bicanic & Kremers, 2007a). The protocol is available in Dutch and Danish. The name STEPS refers to the “step-by-step” approach of the treatment. STEPS primarily aims to reduce PTSD symptomatology.

The main components of STEPS are (1) psychoeducation about rape and its aftermath by psychotherapists as well as medical and forensic professionals, (2) exposure *in sensu* by means of repeated talking and writing about the rape event, (3) cognitive restructuring, (4) exposure *in vivo* (confronting trauma reminders in real life, e.g., situations, objects) to address behavioral avoidance, and (5) relapse prevention. The parallel parents’ support group aims to reduce the parents’ own level of stress and guiding parents in how to successfully support their child by sharing trauma-related feelings, providing psychoeducation, and reframing their own attribution errors. Parents are included in the STEPS protocol based on evidence that parents’ understanding about the impact of events on their children is lacking, in particular on female adolescents, as well as parent–child communication about youth experience of upsetting events and its associated distress (Stover, Hahn, Im, & Berkowitz, 2010). Also, lower levels of parental emotional distress and stronger parental support predict a more positive treatment response (Cohen & Mannarino, 2000). STEPS consists of eight weekly group sessions for four to five subjects, preceded by one preparatory individual session to set treatment goals and to create the *in vivo* hierarchy, and six weekly group sessions for the parents. Each session lasts 120 min. The individual treatment goals come up again in sessions 4–8, when adolescents do assignments of *in vivo* homework. Adolescents who miss more than one session are considered treatment dropouts.

Every subject and every parent (couple) receive a personal exercise book (Bicanic & Kremers, 2007b, 2007c) with information about traumatic stress and coping, and instructions for homework (writing trauma narrative and graded exposure *in vivo*) of 30–60 min weekly. A week-by-week outline of the STEPS protocol is presented in Box 1.

The STEPS protocol is designed to be used in a group format, but can also be applied for individual treatment. The benefits of connecting peers with similar experiences and problems in group treatment include decreasing feelings of isolation and stigmatization, increasing sense of being understood, and establishing a safe and encouraging environment to disclose the trauma narrative. Also, group intervention may be especially appropriate for adolescents who are at a developmental stage in which contact with peers is extremely important.

STEPS is similar to other existing trauma-focused interventions (Cohen et al., 2004; Deblinger et al., 2006), but is unique in the target group: adolescent girls 13–18 years and their parents. STEPS also has some specific characteristics in the inclusion of a police officer and a physician in the therapist team. Additionally, the therapists provide targeted psychoeducation with an emphasis on sexual education and sexual problems. The treatment length of STEPS is nine sessions instead of the usual length in existing TF-CBT protocols (Cohen et al., 2004; Deblinger et al., 2006) of 12–16 sessions.

### Data analyses

In order to evaluate changes in rape-related symptoms over time, a repeated measures analysis of variance (ANOVA) was used. To check the potential influence of “time since trauma,” this variable was included in the repeated measures ANOVA as a covariate. Eta squared (*η* ^2^) is used as effect size, where 0.01 constitutes a small effect, 0.06 a medium effect, and 0.14 a large effect (see Cohen, 1988, p. 283). With concern to the preliminary effectiveness of STEPS, effect sizes (using Cohen’s *d=M*_1_–*M*_2_/s_pooled_, where s_pooled_=√[(s^2^_1_+ s^2^_2_)/2], J.W. Cohen, 1988, pp. 20–27) were calculated for pre- and posttreatment measurements (*t*_*1*_ and *t*_*2*_, respectively), for pre-treatment and 6 months follow-up measurements (*t*_*1*_ and *t*_*3*_, respectively), and for pre-treatment and 12 months follow-up measurements (*t*_*1*_ and *t*_*4*_, respectively). When using the rules of thumb by Cohen (1992) for *d*, we interpret 0.2 as a small effect, 0.5 as a moderate effect, and 0.8 as a large effect. Analyses were performed using SPSS version 17.0.

## Results

### Sample characteristics

STEPS group therapy was applied in 55 adolescent girls and their parents. This group did not differ from the subjects excluded for STEPS group therapy with regard to TSCC scores, age, and time since trauma. The majority of those excluded for STEPS group therapy received EMDR or individual STEPS.

At initial evaluation, the mean age was 16.0 years (*SD*: 1.4 years; range:, 13.5–18.9 years). All girls were living with (one of) their parents, and in 36% of the cases, the biological parents were divorced. Educational track of the girls was lower (58%), middle (21%), and higher (21%) level of secondary school. The rape experience was characterized by completed penetration (oral, vaginal, anal, or combined) in 87% of the adolescents, and 39% of the girls reported physical violence coexisting with the rape. Regarding the identity of the perpetrator, 73% was known to the victim, mostly identified as an (ex-)boyfriend, date, or acquaintance. More than half of the perpetrators (53%) were under the age of 18. The mean time elapsed since trauma was 53.8 weeks (*SD*: 62.3 weeks; median: 26.5 weeks; range: 4–260 weeks). The majority of the victims first disclosed their narrative to a (boy)friend (69%), whereas only 14.5% first disclosed to the parent(s). A small number of adolescents (7%) had previously received trauma-specific treatment. None of the adolescents reported a history of childhood physical abuse or domestic violence. None of the adolescents were currently taking psychotropic medications. The mean score of the rape victims’ SCL-90 total score (*M*=200.31; *SD*=54.2) was comparable with previously reported data of psychiatric populations, *M=*203.55; *SD*=61.60; *t*(5,711)=0.39; *p*=.70, and was substantially higher, *t*(2,421)=18.21; *p*<.001, compared to the general population(*M*=118.28, *SD*=32.38; Arrindell & Ettema, 1986).

### Evaluation of STEPS

#### Changes over time

Across all the four measurements in time, 10.9% were missing data from questionnaires. In Table 1, the results of the repeated measures ANOVA’s are presented, including effect sizes and *p*-values, as well as the descriptive statistics for the four measurements in time on all scales. The results show a significant decline of scores on all subscales over time. Effect sizes, expressed by *η* ^2^, vary between 0.67 for the TSCC-PTS subscale and 0.16 for the TSCC-Anger subscale.

**Table 1:** Results for symptom changes over time (*F*, *p*, *η* ^2^) and effect sizes (Cohen’s *d*) per measurement wave

								Cohen’s *d*,		
								
Scale	*t*_*1*_ *M* (*SD*)	*t*_*2*_ *M* (*SD*)	*t*_*3*_ *M* (*SD*)	*t*_*4*_ *M* (*SD*)	*F* (*df1, df2*)	*P*	*η* ^2^	*t*_*1*_ *–t*_*2*_	*t*_*1*_ *–t*_*3*_	*t*_*1*_ *–t*_*4*_
TSCC										
Anxiety	54.5 (8.4)	48.8 (6.8)	44.1 (8.1)	43.0 (7.9)	28.4 (2.5, 100.2)	<.001	.42	0.75	1.26	1.41
Depression	52.8 (8.9)	46.3 (7.3)	42.8 (8.5)	42.3 (7.9)	34.0 (2.4, 97.3)	<.001	.46	0.80	1.15	1.25
PTS	59.9 (6.6)	50.0 (7.5)	44.2 (8.1)	42.6 (6.4)	80.8 (2.3, 100.0)	<.001	.67	1.40	2.13	2.66
Anger	44.0 (7.3)	42.1 (6.3)	39.9 (6.3)	39.7 (6.5)	7.3 (2.2, 86.4)	<.05	.16	0.28	0.60	0.62
Dissociation	51.6 (8.0)	46.2 (6.6)	42.7 (7.5)	42.9 (7.3)	24.0 (2.2, 88.8)	<.001	.38	0.74	1.15	1.14
Sexual concerns	53.6 (11.0)	50.6 (9.5)	43.4 (6.4)	43.5 (7.6)	21.1 (2.3, 91.9)	<.001	.35	0.29	1.13	1.07
YSR										
Internalizing	64.6 (7.1)	55.0 (9.5)	49.8 (12.6)	47.0 (12.4)	38.9 (3, 117)	<.001	.50	1.14	1.45	1.74
Externalizing	54.7 (8.0)	52.3 (9.2)	49.9 (9.8)	48.0 (10.1)	12.0 (3, 117)	<.001	.24	0.28	0.54	0.74
Total	60.6 (6.3)	53.3 (8.6)	49.2 (11.6)	46.2 (11.2)	37.2 (2.5, 97.0)	<.001	.49	0.97	1.22	1.58

*Note*. Number of complete cases, *n*=41. TSCC=Trauma Symptom Checklist for Children (*t*-scores subscales range: anxiety 35–92; depression 36–85; posttraumatic stress 35–82; anger 35–78; dissociation 36–90; sexual concerns 36–175); YSR=Youth Self-Report (*t*-scores subscales range: internalizing 50–100; externalizing 50–100; total 50–100). *t*_*1*_=pre-treatment, *t*_*2*_=posttreatment, *t*_*3*_=6 months follow-up, and *t*_*4*_=12 months follow-up. *M*=mean, *SD*=standard deviation.

**Box 1:** Session-by-session outline of STEPS protocol, girls’ and parents’ group.

Session-by-session outline of the STEPS protocol, girls’ group	
Session 0	Setting treatment goals
Session 1	Group rules, rationale STEPS, exposure *in sensu* by trauma narrative (1)
Session 2	Trauma narrative (2), education rape, understanding cognitive triangle
Session 3	Trauma narrative (3), rationale graded exposure *in vivo*
Session 4	Graded exposure *in vivo* (1), education sex and sexual problems
Session 5	Graded exposure *in vivo* (2), visit by physician
Session 6	Graded exposure *in vivo* (3), education revictimization and future safety
Session 7	Graded exposure *in vivo* (4), visit by police officers
Session 8	Graded exposure *in vivo* (5), relapse prevention, evaluation
Session-by-session outline of the STEPS protocol, parents’ group	
Session 1	Sharing trauma narrative, education (impact of) rape, rationale STEPS
Session 2	Understanding cognitive triangle after rape
Session 3	Dysfunctional cognitions of parents
Session 4	Education sexual problems, visit by physician
Session 5	Education revictimization, visit by police officers
Session 6	Relapse prevention, evaluation

We found no significant interaction-effect between “time since trauma” and the four measurements in time for all scales (*p*-values ranging between 0.31 and 0.86, and *η* ^2^ ranging between 0.005 and 0.031). As “time since trauma” was not a significant covariate in all other cases, it was left out of the reported analyses reported in Table 1.

Effect sizes of STEPS per measurement wave, expressed by Cohen’s *d*, can be found in the final two columns of Table 1. Cohen’s *d* was calculated for the effect of difference in symptomatology between pre-treatment and, respectively, posttreatment (*t*_*1*_–*t*_*2*_), 6 months follow-up (*t*_*1*_–*t*_*3*_), and 12 months follow-up (*t*_*1*_–*t*_*4*_). The effect sizes for *t*_*1*_ and *t*_*2*_ ranged from moderate to large effects (*d* between 0.28 and 1.40). We found large effects for all scales for the difference in symptomatology between *t*_*1*_ and *t*_*3*_ (*d* between 1.13 and 2.13), except for anger of the TSCC (where we found a moderate effect of 0.60) and externalizing problems of the YSR (where we found a moderate effect of 0.54). We found large effects for all scales for the difference in symptomatology between *t*_*1*_ and *t*_*4*_ (*d* between 1.07 and 2.66), except for anger of the TSCC (where we found a moderate effect of 0.62) and externalizing problems of the YSR (where we found a moderate effect of 0.74). Again, the smallest effect was found for Anger of the TSCC and Externalizing problems of the YSR. The largest effect was found for PTS of the TSCC.

#### Dropout and attendance

All subjects, except for one (1.8%), completed the STEPS treatment. Of the completers, 72.2% (*n*=39) attended all eight sessions, and 27.8% (*n*=15) attended seven out of eight sessions. In Fig. 1, dropouts during follow-ups are presented. At 6-month follow-up, the researchers accidentally neglected the administration of questionnaires in one treatment group of four subjects. At 12-month follow-up, questionnaires from a total of nine subjects were missing due to refusal to participate in follow-up (5), problems with tracing subjects (2), stay in hospital because of voluntary pregnancy (1), and stay in an inpatient clinic (1). No significant difference was found between 12-month follow-up subjects and noncompleters on pre-treatment level of symptoms.

#### Clinical relevance

For the assessment of clinical relevance, we calculated the percentages of subjects who still scored within (sub)clinical ranges on the TSCC-PTS (range: 50–82) on the last follow-up compared to pre-treatment. At 12-month follow-up, no subject scored in the clinical range compared to 11 subjects (20%) at pre-treatment. Also, seven subjects (12.7%) scored in the subclinical range on the TSCC-PTS subscale at 12-month follow-up, compared to 44 subjects (80%) at pre-treatment.

## Discussion

After completing STEPS, victims of single rape reported a significant decrease in rape-related symptoms. The largest decrease occurred in PTS symptoms between pre- and posttreatment, and this gain was maintained at 12-month follow-up. The smallest decrease occurred in externalizing problems. The treatment improvement appears to be statistically as well as clinically significant in that subjects’ scores on standardized measures reached or approached normal ranges on multiple dimensions.

STEPS is specifically designed for rape victims, but similar to other established and proven effective programs, notably (group-based) TF-CBT developed in the USA (Cohen et al., 2004; Deblinger et al., 2006) with respect to basic elements such as exposure *in sensu* and *in vivo*, cognitive restructuring, and psychoeducation. Minor differences are related to targeted age group, treatment length, the involvement of medical and forensic professionals in the program, and the focus on sexual education including (prevention of) sexual problems. Focusing on the latter problems is important as there is some evidence suggesting that the experience of rape during adolescence increases the risk of sexual problems in later adult life (Postma, Bicanic, Van der Vaart, & Laan, 2013).

The results of the present study in a well-described homogenous patient group with high compliance rates and long-term follow-up suggest that STEPS may be a promising treatment protocol for adolescents with rape-related symptomatology. The effect sizes were large and comparable to those in prior studies on children with sexual abuse-related PTSD symptoms (Cohen et al., 2004; Deblinger et al., 2006). The finding that time since trauma was not a significant covariate in the outcome, may indicate that the decrease in symptomatology might not simply be due to the passing of time or natural course, but might be attributable to the STEPS intervention. However, with a median of time since trauma of 26.5 weeks, half of the sample was treated during a time when natural recovery may have occurred. Therefore, the encouraging findings need confirmation in future controlled studies on the effectiveness of STEPS because it may be possible that the treatment works especially well for more chronic symptoms, while the less chronic part of the sample showed considerable improvement on its own. Then again, rape is known as a traumatic event with the highest risk for PTSD (Kessler et al., 1995). A prospective study in adult victims of rape showed that 94% reported severe symptoms of PTS after 1 week (Rothbaum et al., 1992). This percentage decreased naturally to 65% after 1 month and to 47% after 3 months. Although some victims will recover spontaneously in the time-period between 1 and 3 months, our sample was actively seeking professional help suggesting problems with the natural recovery process.

Although one-third of the subjects who were offered treatment preferred an individual setting, it is noteworthy that 54 out of the 55 subjects who preferred a group setting completed STEPS with high attendance. These results support STEPS as a highly feasible and acceptable treatment approach for adolescents and their parents. The low attrition rate was somewhat surprising given the fact that adolescents are not easy to engage, the high intensity of the intervention, and average attrition rates of 20% reported in the literature regarding PTSD interventions (Hembree et al., 2003; Vickerman & Margolin, 2009). The low attrition rate may be attributed to (1) high group cohesion because of the small size of the groups; (2) the limited number of sessions; (3) the presence of a parallel parents’ group, increasing the pressure for adolescents to attend; (4) the highly structured protocol; (5) the high functioning sample as all adolescents were attending school and living with their parent(s); and (6) the highly motivated and dedicated therapists.

Several caveats should be noted. STEPS has been developed for the group of adolescents with psychological problems after single rape, but without prior sexual abuse. This group my not reflect a general group of rape victims, as rape is strongly related to a history of childhood sexual abuse and to sexual revictimization (Maker, Kemmelmeier, & Peterson, 2001). Additionally, the results cannot be generalized to female adolescents who are victimized by chronic or multiple sexual abuse. Another limitation of the study is that treatment fidelity was not assessed nor was the benefit of the parental involvement. Despite the lack of information on the benefit of the parent support group, we recommend involving parents in trauma-focused treatments because parental stress predicts PTS in children (Alisic, Jongmans, Van Wesel, & Kleber, 2011). Parents are educated about PTSD symptoms that are difficult to observe, such as avoidance and re-experiencing. Without explanation on stress symptoms, parents may misperceive behavior changes as part of normal adolescence.

Based on (inter)national reports showing that adolescence is a life-time period of increased risk for sexual assault (De Haas, Van Berlo, Bakker, & Vanwesenbeeck, 2012; Tjaden & Thoennes, 2006), as well as a period in life in which sexual assault can have devastating effects on the individual, it is important to identify effective treatment programs. STEPS may be one possible option to help adolescents and their parents to recover from the impact of single rape, especially because it seems a highly feasible CBT-based intervention. CBT is known as the first treatment of choice for PTSD in children and youth (National Institute for Clinical Excellence, 2005), but in recent years the effectiveness of EMDR for children is also revealed in more than 15 studies (Fleming, 2012). To best assess the (relative) effectiveness of STEPS, (individual) STEPS should be compared to another exposure-based treatment condition such as EMDR in a randomized study design. High-quality research with appropriate study designs is required to confirm the promising results from the current study and to ascertain the most effective and time-efficient treatment for this population, that is at high risk for revictimization (Humphrey & White, 2000).

## References

[CIT0001] Achenbach T. M, Rescorla L. A (2001). Manual for the ASEBA school-age forms and profiles.

[CIT0002] Alisic E, Jongmans M. J, Van Wesel F, Kleber R. J (2011). Building child trauma theory from longitudinal studies: A meta-analysis. Clinical Psychology Review.

[CIT0003] Arrindell W. A, Ettema J. H. M (1986). SCL-90: Handleiding bij een multidimensionele psychopathologie-indicator [SCL-90 symptom checklist: Manual to a multidimensional psychopathology-indicator].

[CIT0004] Avinger K. A, Jones R. A (2007). Group treatment of sexually abused adolescent girls: A review of outcome studies. The American Journal of Family Therapy.

[CIT0006] Bal S, Uvin K (2009). De Trauma Symptom Checklist als screeningsinstrument voor trauma-symptomatologie bij adolescenten: psychometrische kwaliteiten van de Nederlandse vertaling [The Trauma Symptom Checklist as a screening instrument for trauma-symptomatology in adolescents: psychometric properties of the Dutch translation]. Gedragstherapie.

[CIT0007] Bérubé R. L, Achenbach T. M (2006). Bibliography of published studies using ASEBA instruments: 2006 edition.

[CIT0008] Bicanic I, Kremers A (2007a). STEPS: Cognitieve gedragstherapie bij Post Traumatische Stress Stoornis na eenmalig seksueel geweld. Therapeutenhandleiding [STEPS: Cognitive Behavior Therapy for Post Traumatic Stress Disorder due to sexual violence. Manual].

[CIT0009] Bicanic I, Kremers A (2007b). STEPS: Cognitieve gedragstherapie bij Post Traumatische Stress Stoornis na eenmalig seksueel geweld. Werkboek meisje [STEPS: Cognitive Behavior Therapy for Post Traumatic Stress Disorder due to sexual violence. Workbook adolescent].

[CIT0010] Bicanic I, Kremers A (2007c). STEPS: Cognitieve gedragstherapie bij Post Traumatische Stress Stoornis na eenmalig seksueel geweld. Werkboek ouder(s) [STEPS: Cognitive Behavior Therapy for Post Traumatic Stress Disorder due to sexual violence. Workbook parent].

[CIT0011] Bicanic I, Postma R, Sinnema G, De Roos C, Olff M, Van De Putte E (2013). Salivary cortisol and dehydroepiandrosterone sulfate in adolescent rape victims with post traumatic stress disorder. Psychoneuroendocrimology.

[CIT0012] Briere J (1996). Trauma Symptom Checklist for Children (TSCC).

[CIT0013] Cohen J (1992). A power primer. Psychological Bulletin.

[CIT0014] Cohen J. A, Deblinger E, Mannarino A. P, Steer R. A (2004). A multisite, randomized controlled trial for children with sexual abuse-related PTSD symptoms. Journal of the American Academy of Child and Adolescent Psychiatry.

[CIT0015] Cohen J. A, Mannarino A. P (2000). Predictors of treatment outcome in sexually abused children. Child Abuse and Neglect.

[CIT0016] Cohen J. W (1988). Statistical power analysis for the behavioral sciences.

[CIT0017] Deblinger E, Mannarino A. P, Cohen J. A, Steer R. A (2006). A follow-up study of a multisite, randomized, controlled trial for children with sexual abuse-related PTSD symptoms. Journal of the American Academy of Child and Adolescent Psychiatry.

[CIT0018] De Haas S, Van Berlo W, Bakker F, Vanwesenbeeck I (2012). Prevalence and characteristics of sexual violence in the Netherlands, the risk of revictimization and pregnancy: Results from a national population survey. Violence and Victims.

[CIT0019] Fleming J (2012). The effectiveness of eye movement desensitization and reprocessing in the treatment of traumatized children and youth. Journal of EMDR Practice and Research.

[CIT0020] Hansen M, Armour C, Elklit A (2012). Assessing a dysphoric arousal model acute stress disorder symptoms in a clinical sample of rape and bank robbery victims. European Journal of Psychotraumatology.

[CIT0021] Hembree E. A, Foa E. B, Dorfan N. M, Street G. P, Kowalski J, Tu X (2003). Do patients drop out prematurely from exposure therapy for PTSD?. Journal of Traumatic Stress.

[CIT0022] Humphrey J. A, White J. W (2000). Women’s vulnerability to sexual assault from adolescence to young adulthood. Journal of Adolescent Health.

[CIT0023] Kessler R. C, Sonnega A, Bromet E, Hughes M, Nelson C. B (1995). Posttraumatic stress disorder in the National Comorbidity Survey. Archives of General Psychiatry.

[CIT0024] Lange A (2004). Vragenlijst Seksuele rauma’s in het verleden: VST-V: Een anamnestisch instrument voor onderzoek en praktijk: handleiding en verantwoording [Childhood unwanted sexual events (CHUSE): Manual].

[CIT0025] Maker A. H, Kemmelmeier M, Peterson C (2001). Child sexual abuse, peer sexual abuse, and sexual assault in adulthood: A multi-risk model of revictimization. Journal of Traumatic Stress.

[CIT0026] McLaughlin K. A, Koenen K. C, Petuhkova M, Sampson N. A, Zaslavsky A. M, Kessler R. C (2013). Trauma exposure and posttraumatic stress disorder in a national sample of adolescents. Journal of the American Academy of Child and Adolescent Psychiatry.

[CIT0027] National Institute for Clinical Excellence (2005). Posttraumatic Stress Disorder (PTSD): *The management of PTSD in adults and children in primary and secondary care, report nr 26*.

[CIT0028] Postma R, Bicanic I, Van der Vaart H, Laan E (2013). Pelvic floor muscle problems mediate sexual problems in young adult rape victims. The Journal of Sexual Medicine.

[CIT0029] Risser H. J, Hetzell-Riggin M. D, Thomsen C. J, McCanne T. R (2006). PTSD as a mediator of sexual revictimization: The role of reexperiencing, avoidance, and arousal symptoms. Journal of Traumatic Stress.

[CIT0030] Rothbaum B. O, Astin M. C, Marsteller F (2005). Prolonged exposure versus eye movement desensitization and reprocessing (EMDR) for PTSD rape victims. Journal of Traumatic Stress.

[CIT0031] Rothbaum B. O, Foa E. B, Riggs D. S, Murdock T, Walsh W (1992). A prospective examination of post traumatic stress disorder in rape victims. Journal of Traumatic Stress.

[CIT0032] Siebelink B. M, Treffers D. A (2001). Anxiety disorder interview schedule for DSM-IV—Child version; Dutch version.

[CIT0033] Silverman W. K, Albano A. M (1996). Anxiety disorder interview schedule for DSM-IV child version, child interview schedule.

[CIT0034] Stover C, Hahn H, Im J, Berkowitz S (2010). Agreement of parent and child reports of trauma exposure impact and symptoms in the early aftermath of trauma. Psychological Trauma: Theory Research Practice and Policy.

[CIT0035] Tjaden P, Thoennes N (2006). Extent, nature and consequences of rape victimization: Findings from the National Violence Against Women Survey.

[CIT0036] Verhulst F. C, Van der Ende J, Koot H. M (1997). Handleiding voor de YSR [Manual for the YSR].

[CIT0037] Vickerman K. A, Margolin G (2009). Rape treatment outcome research: Empirical findings and state of the literature. Clinical Psychology Review.

